# Dentist´s attitude and criteria in the diagnosis and treatment of caries lesions: Survey about a clinical case

**DOI:** 10.4317/jced.58364

**Published:** 2022-01-01

**Authors:** Sebastiana Arroyo-Bote, Susane Herrero-Tarilonte, Joan Mas-Ramis, Catalina Bennasar-Verger

**Affiliations:** 1 Coordinating teacher. Conservative Dentistry. ADEMA University School. University of the Balearic Islands. Spain. Associate professor Barcelona University. IDIBELL research; 2 Associate professor Conservative Dentistry. ADEMA University School. University of the Balearic Islands. Spain

## Abstract

**Background:**

The diagnosis of caries, the stage of the disease and the indication of treatment are among the most controversial issues in dentistry. Studies comparing the diagnosis and treatment indication of different professionals show the lack of a unifying criterion in the diagnosis and treatment plan of the disease. The Objectives of this research is to evaluate the attitude of a group of odontostomatologists to a clinical case with lesions compatible with caries, evaluating whether their criteria and attitude in diagnosis and treatment depend on their academic level, years of professional practice, the application of minimal intervention dentistry criteria and the usual practice in conservative restorative treatments.

**Material and Methods:**

A survey was applied to dentists registered at the Illustrious Official College of Dentists of the Balearic Islands. The questionnaire was developed by the researchers from a real clinical case. A descriptive statistical analysis was performed of all the generated data and, to evaluate the association between the survey responses and the variables of interest, the χ^2 of independence test was performed. In addition, tests comparing the corresponding proportions were conducted using Fisher’s exact test.

**Results:**

Regarding pit and fissure significant differences were found in the diagnosis in 46 in terms of dentists’ qualifications and in the treatment between the application of minimal intervention dentistry criteria and the usual practice in conservative restorative treatments. No significant differences were found in the other variables analyzed. As regards caries lesions on proximal surfaces, no significant differences were found in the diagnosis or treatment in any of the variables analyzed.

**Conclusions:**

That there is no change in the professional attitude towards the diagnosis and treatment of caries lesions in this group of professionals, having very interventionist criteria and attitudes in all variables analyzed.

** Key words:**Atraumatic restorative treatment, caries detection, demineralization, non-cavitated caries lesions, radiography.

## Introduction

The diagnosis of caries, the stage of the disease and the indication of treatment are among the most controversial issues in dentistry. Classically, the diagnosis of caries lesions is made through visual inspection, tactile exploration, and radiographic examination. Studies comparing the diagnosis and treatment indication of different professionals show the lack of a unifying criterion in the diagnosis and treatment plan of the disease. The classic treatment of clinically detectable caries lesions is the total elimination of demineralized tissues and their replacement with filling materials. However, scientific evidence has opened up new lines of treatment for caries lesions, demonstrating the benefits of treatments focused on the prevention and remineralization of the lesions with minimal intervention to stop the progression of the disease and preserve as much dental tissue as possible (1).

Prevention of the disease can be done in three stages.

1. Primary stage: Before the disease is present or clinically detectable.

2. Secondary stage: When the disease is present and can be clinically detected with non-cavitated lesions.

3. Tertiary stage: When the disease is clinically detectable with cavitated lesions.

Primary prevention is based on patient education, diet control, application of fluoride and preventive sealants to avoid the development of the disease, tools that also serve for secondary prevention (1) where remineralization treatments and sealants play a very important role in the evolution and control of non-cavitated demineralized caries lesions, as advised by the International Caries Detection and Assessment System since 2002 (2,3). It is in the face of this secondary prevention that caries classification and lesion activity are very important in establishing the best treatment strategy (4-6) Tertiary prevention encompasses all the tools for Fighting the disease, from restorative treatment of cavitated lesions to the strategies used in primary and secondary prevention, since the patient clearly manifests the presence of the disease in its most advanced stages. It is also at this stage that the attitude of clinicians towards cavitated lesions should be focused on minimally invasive treatments with non-invasive or microinvasive treatments (7) Also noteworthy in invasive treatments are the new criteria in the removal of carious dentin (8) and the timing of the intervention through invasive dental restoration treatments with Black’s cavity preparations.

In occlusal cavitated lesions and/or proximal faces, visual inspection and tactile exploration are the most commonly used diagnostic methods. In non-cavitated lesions, transillumination and radiographic study are very important for the detection of lesions. The industry has developed diagnostic methods that help the clinician in decision-making, especially in early lesions that are difficult to detect, where diagnostic systems based on tooth autofluorescence (such as QLF) and electrical resistance (such as ECM) seem to offer the best hope for reliable and accurate detection of the early stages of enamel demineralization (9). However, in order to act in very early stages, it is necessary to have diagnostic methods that can detect the disease in the early stages.

There is a changing paradigm in caryology where we are slowly moving away from a surgical model to a medical one. Devices that allow early caries detection make it easier for remineralizing therapies to be correctly indicated (9). It is evident that the management of caries lesions has changed in recent decades, recommending early diagnosis and non-invasive or microinvasive treatments. However, these scanning techniques are not popular among dentists, so few have these scanning methods.

However, despite the preventive and minimally invasive approaches that the scientific evidence shows in this disease, the attitude of the clinician remains highly interventionist with great disparity in criteria, which has led some scientific societies to develop intervention protocols, such as the Japanese Conservative Dentistry Society, which developed a guide for the unification of diagnostic and treatment criteria based on scientific evidence (10).

The aim of this study is to discern the attitude of a group of dentists to the same clinical case. The objective is to know the differences in the diagnoses and in the indication of the treatments.

Main objective: To evaluate the diagnosis and treatment of a sample of odontostomatologists in the Balearic Islands in the resolution of a clinical case with compatible caries lesions.

Secondary objectives: To study if there is a link between the diagnosis and treatment of the lesions and the time in professional practice, degree, habitual practice of restorative dentistry and application of minimal intervention dentistry.

Hypothesis.

-The odontostomatologist’s time in professional practice influences the diagnosis and treatment indicated in teeth with lesions compatible with caries.

-The odontostomatologist’s qualifications influence the diagnosis and treatment indicated in teeth with lesions compatible with caries.

-The odontostomatologist’s daily practice of restorative treatments influences the diagnosis and treatment indicated in teeth with lesions compatible with caries.

-The odontostomatologist’s concept of minimally invasive treatments influences the treatment indicated in teeth with lesions compatible with caries.

## Material and Methods

This investigation complies with the Helsinki Declaration, and the patient signed an informed consent form. The study has the approval of the Ethics Committee of the Balearic Islands, with research project nº. IB4142/20PI.

A survey was applied to dentists registered at the Illustrious Official College of Dentists of the Balearic Islands. The survey was mailed to members from the secretary’s office of the professional association, using a form created with www.surveymonkey.com. There were 659 participants.

**Figure 1:**
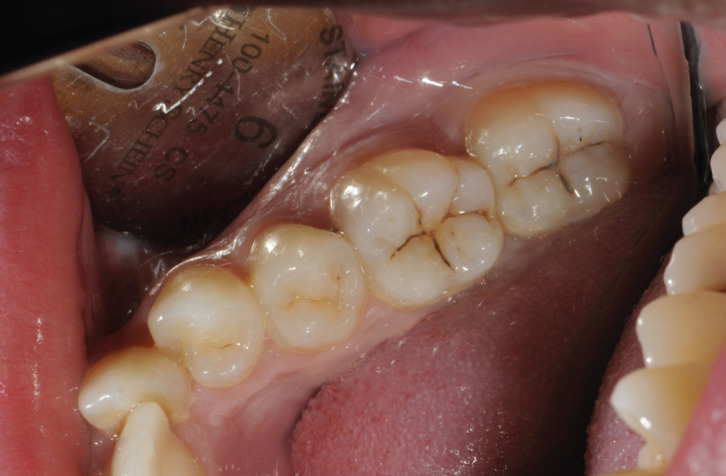
Intraoral image of the clinical case evaluated in the survey. The posterior sector of the fourth quadrant can be seen. (44, 45, 46 and 47).

**Figure 2:**
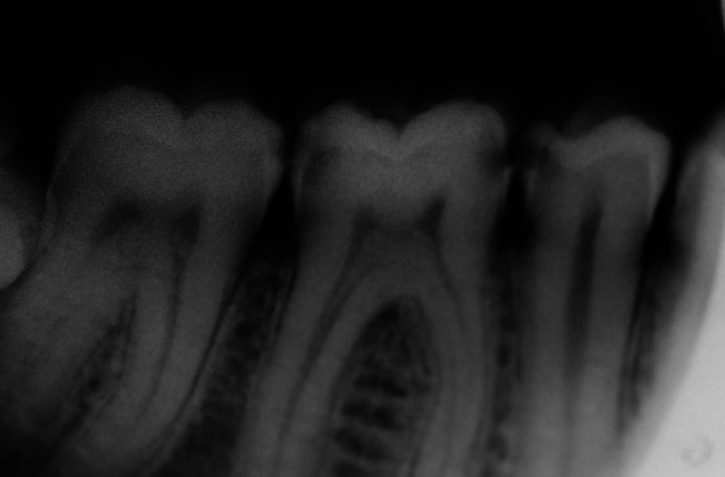
Radiographic image of the clinical case evaluated in the survey. The posterior sector of the fourth quadrant can be seen. (44, 45, 46 and 47).

The questionnaire was developed by the researchers from a real clinical case (Figs. 1,2).

The survey consists of 11 questions, which are placed in three groups:

1. Questions 1-3 address fossa and fissure injuries (Fig. 1).

2. Questions 4-7 address the lesions on the proximal faces (Fig. 2).

3. Questions 8-11 address the characteristics of the respondent.

The survey was sent by mail, through a link to registered dentists in the Illustrious Official College of Dentists of the Balearic Islands in February 2020. It was open for a month. It was answered by 20.33% of registered dentists. All the surveys received were complete, and no technical problem was detected, so no survey received was invalidated, including 100% of the surveys answered in the study, in total 134.

A descriptive analysis was performed of all the generated data and, to evaluate the association between the survey responses and the variables of interest, the χ^2 of independence test was performed. In addition, in the cases where there were differences that could be important between the proportions of the extreme groups (for example, less than 5 years vs. more than 30 or doctor vs. degree), tests comparing the corresponding proportions were conducted using Fisher’s exact test.

## Results

The survey was answered by 134 dentists. The results are shown in Table 1,  Table 1 cont., where all the questions are represented with the descriptive statistical analysis of the data. 82.5% of the respondents recognized that they applied concepts of minimal intervention, 9.5% stated that they did not apply concepts of minimal intervention and 7.9% did not know/no response. Most of the respondents have been practicing for 5-15 years 45.2%. The 92.8% practice conservative dentistry on a daily basis. 34.1% have a master’s degree, 26.9% have a postgraduate degree, only 4.7% are doctors.

**Table 1:**
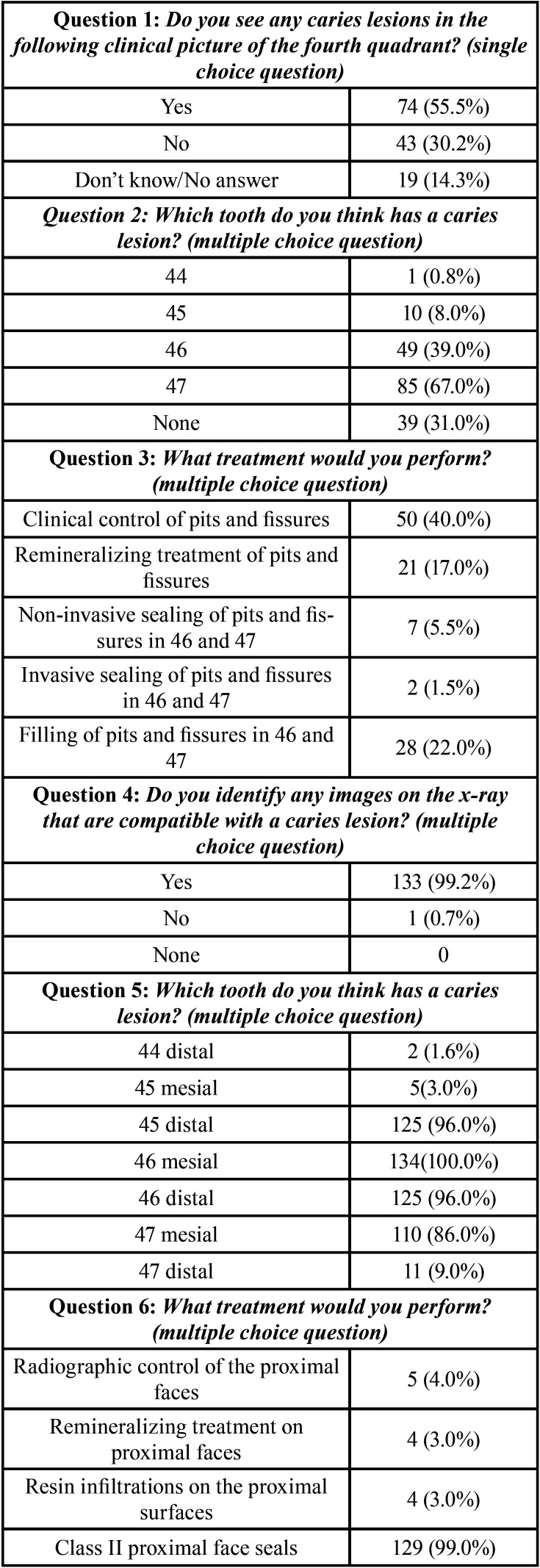
Descriptive statistical result of the survey.

**Table 1 cont.**
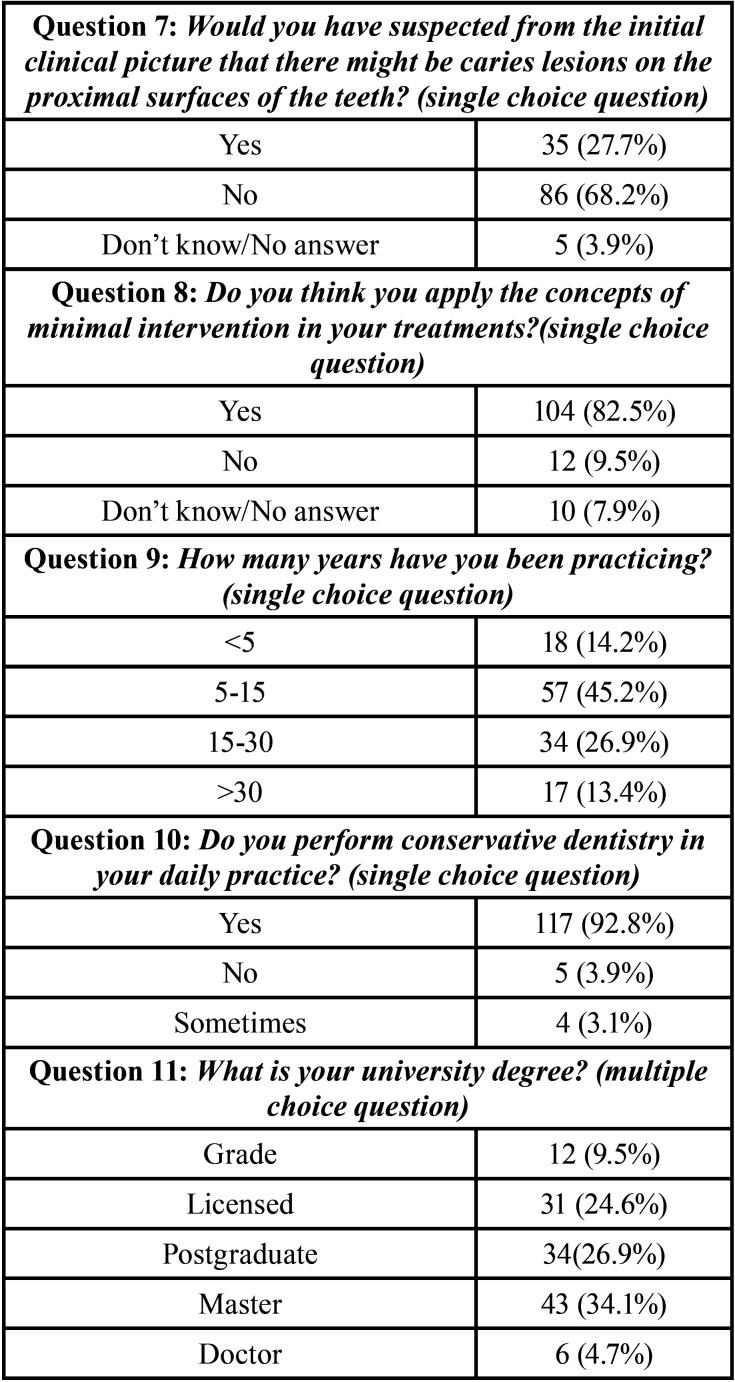
Descriptive statistical result of the survey.

Table 2 shows the results of diagnosis and treatment of caries-compatible lesions in pits and fissures (Fig. 1: Clinical image), relating the years of professional practice and academic qualifications.

**Table 2:**
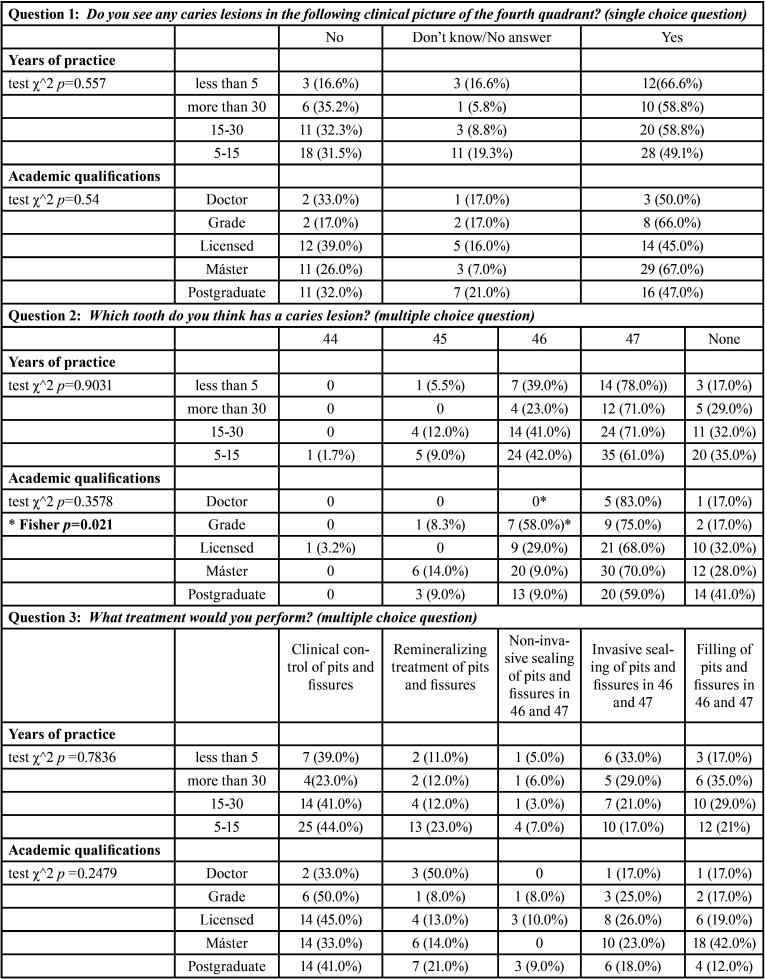
Relationship between diagnosis and treatment of clinical picture with years of professional practice and academic qualifications.

Table 3 shows the results of diagnosis and treatment of caries-compatible lesions on proximal surfaces (Fig. 2: Radiographic image), relating the years of professional practice and academic qualifications.

**Table 3:**
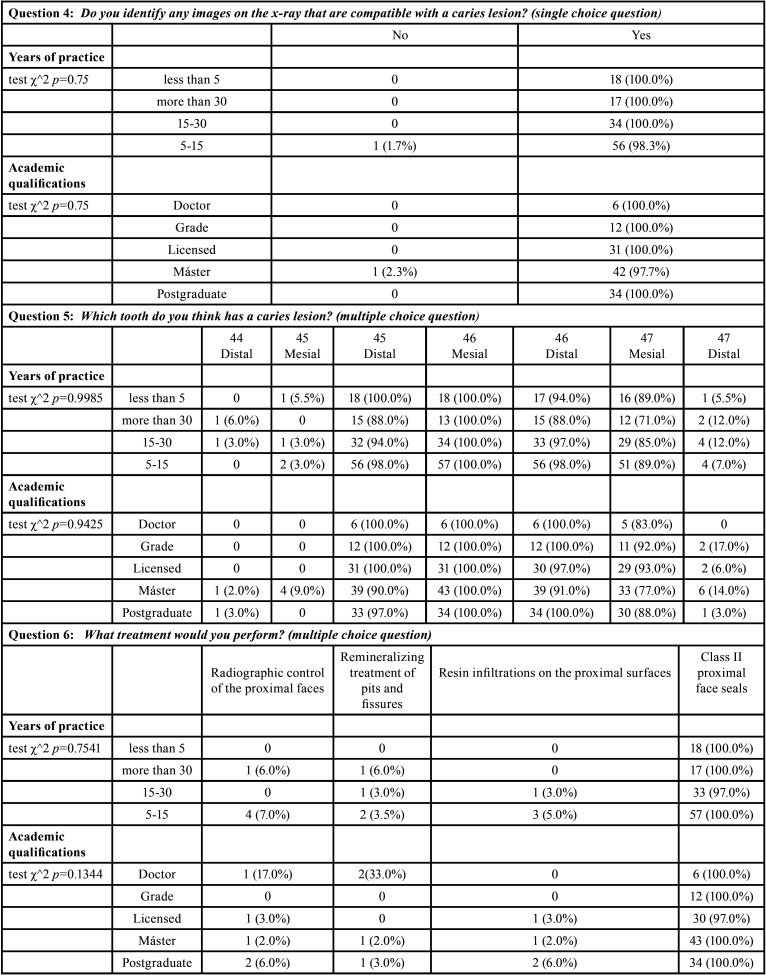
Relationship between diagnosis and treatment of radiographic imaging with years of professional practice and academic qualifications.

In Table 4, the results of questions (3 and 6) referring to treatment of both lesions compatible with pit and fissure caries and proximal surfaces are presented, relating the application of concepts of minimal intervention and the daily practice of conservative treatment with the indications for treatment.

**Table 4:**
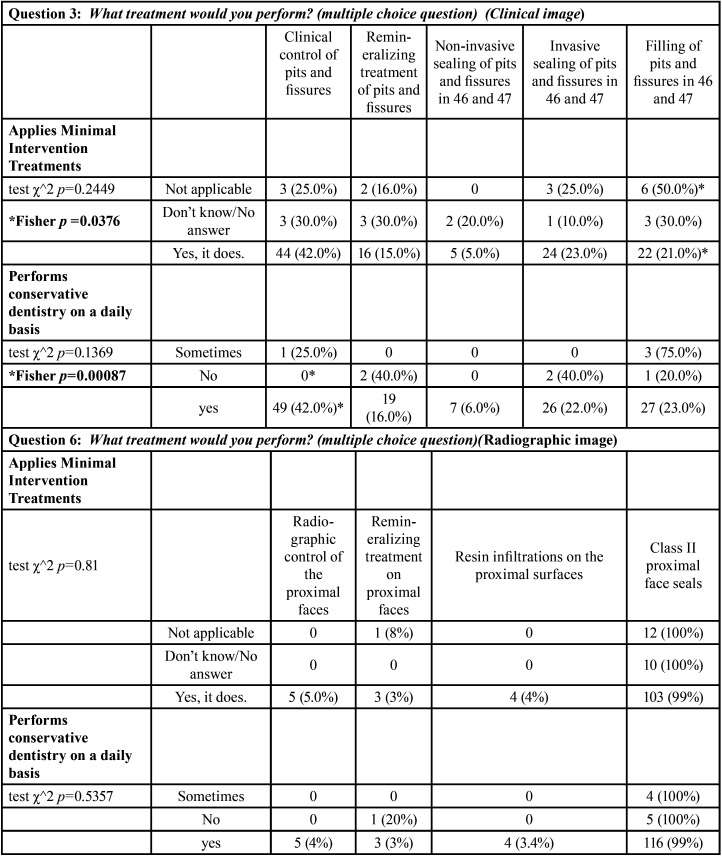
Relationship between clinical and radiographic imaging treatment with the application of minimal intervention concepts and the implementation of conservative treatments in daily practice.

Regarding the diagnosis of caries in pits and fissures, 55% detected caries lesions in the clinical image (Fig. 1), and 30.2% did not. In the statistical analysis, no significant differences were found in relation to the years of professional practice (*p-value* = 0.557) or to professional qualification (*p-value* = 0. 54). 67% located the lesion in 47 compared to 39% who located it in 46. No significant differences were found in any of the variables except in the assessment of 46 where Fisher’s exact test for comparison of proportions was used; in this case, the difference between the percentages in the “Degree” group and the “Doctorate” group was significant (*p-value*=0.021) (Table 2).

As regards the indicated treatment, no significant differences were found in relation to the years of professional practice (*p-value* = 0.7836) or academic qualification (*p-value* = 0.2479) (Table 2).

In terms of the indicated treatment and the application of minimal intervention concepts, significant differences were found in the indication of filling in pits and fissures in teeth 46 and 47 between the group that applied and the group that did not apply such minimal interventions (Fisher’s exact test *p-value* = 0.0376). Significant differences were also found between the group that did perform daily conservative dentistry practice and the group that did not in the treatment indicated as clinical control of the lesions (Fisher’s exact test *p-value* = 0.00087). The remaining treatments and variables analyzed did not show any significant differences (Table 4).

Regarding proximal lesions (Fig. 2), 99.2% recognized caries lesions. There were no significant differences in relation to years of professional practice (*p-value* = 0.75) or to academic qualification (*p-value* = 0.75). There were also no significant differences in the location of the lesions between years of professional practice (*p-value*=0.9985) and the respondent’s academic degree (*p-value*=0.9425) (Table 3). According to the χ^2 test, no association was detected between the treatment indicated in proximal lesions and the application of minimal intervention in the treatments (*p-value*=0. 81) nor with the performance of conservative dentistry in daily practice (*p-value*=0.5357) (Table 4).

68.2% of the respondents had not suspected the presence of lesions in nearby areas with the clinical picture of the case.

## Discussion

Caries is the most prevalent disease in humans and one of the oldest. There are multiple terms and variations in diagnostic classifications and treatment indications for the disease. The European Organization for Caries Research (ORCA) and the Cariology Research Group of the International Association for Dental Research (IADR Cariology Research) organized a workshop that brought together sixteen cariology experts who worked for two days to identify and select the most used terms in dental caries and dental caries management and define them based on current concepts (11). Fifty-five terms were discussed and a consensus was reached on 17 terms, four of which were related to this research:

Definitions of Dental Caries as a Disease (100%): Dental caries is a biofilm-mediated, diet modulated, multifactorial, non-communicable, dynamic disease resulting in net mineral loss of dental hard tissues [Fejerskov 1997; Pitts *et al*., 2017]. It is determined by biological, behavioral, psychosocial, and environmental factors. As a consequence of this process, a caries lesion develops.

Caries Diagnosis (94%): Caries diagnosis is the clinical judgement integrating available information, including the detection and assessment of caries signs (lesions), to determine presence of the disease. The main purpose of clinical caries diagnosis is to achieve the best health outcome for the patient by selecting the best management option for each lesion type, to inform the patient, and to monitor the clinical course of the disease [Nyvad *et al*., 2015].

Caries Care/Management/Control (100%): Caries care/management/control are actions taken to interfere with mineral loss at all stages of the caries disease [Nyvad and Fejerskov, 2015], including non-operative and operative interventions/treatments. Because of the continuous de/remineralization processes, caries control needs to be continued throughout the life course. The terms caries care/management/control may be more appropriate than the term Caries prevention.

Minimal Intervention Dentistry (81%): Minimal intervention dentistry is a holistic caries management philosophy that integrates caries lesion control and minimal operative intervention. The main objective is tissue preservation, including early caries detection and non-operative treatment, combined with minimally invasive restorative procedures [Frencken *et al*., 2012].

The first challenge for the clinician in the face of caries disease is the diagnosis of the lesions. In the clinical case evaluated in this study, the posterior sector of the fourth quadrant can be seen, with staining in the pits and fissures of 46 and 47 (Fig. 1). No cavitated lesions are evident on any of the teeth. For 55.5% of those surveyed, these stains were sufficient to indicate the existence of caries lesions, 30.2% did not identify them as caries and 14.3% did not know whether they were caries or not. In tooth 46, significant differences are demonstrated between the diagnosis of caries in graduate dentists and doctors. This disparity of criteria highlights the difficulty of diagnosis in non-cavitated lesions where there is no evidence of demineralization of dental tissues on the occlusal surfaces. If we analyze the radiographic image of this clinical case (Fig. 2), we can see that there is no effect on the dentin or signs of demineralization in this area in any tooth; hence, if there is any structural alteration in the enamel of the pits and fissures, it is in stages not clinically detecTable by visual inspection. It is in these situations where complementary diagnostic methods such as quantitative light-induced fluorescence and digital image fiber optic transillumination (9,12), can help us to identify and clinically monitor very early lesions that should be treated non-invasively with the aim of stopping the progression of the disease and remineralizing the affected tissues. The research carried out on French general dentists by Doméjean *et al*. (13) in 2012 agree with our results regarding the discrepancy in decision-making in the management of caries in pits and fissures. Those authors concluded that this behavior was already demonstrated in 2002 and persisted in 2012, with an interventionist attitude in lesions that could benefit from non-invasive treatments, although there was some variation according to the dentist’s age, sex, and training in cariology. Another study carried out in 2016 on Norwegian dentists showed a lower tendency to invasive behaviors in occlusal lesions limited to enamel in 2009 (12%) compared to 1995 (18%), with young dentists having a less interventionist attitude. In our results, 22% of the respondents opted for invasive treatments, and no significant differences were found in relation to the years of professional practice (test χ ^ 2 *p* = 0.7836)

Carious lesion activity is an important component that must be considered when making decisions about the appropriate clinical management of caries. The development and use of validated techniques that are easy to use in daily dental practice is important (14). Likewise, the patient’s risk of caries is one of the parameters that must be assessed when making clinical decisions, especially in the case of early caries lesions. One of the most widely used tools for assessing caries risk is the CAMBRA method. However, the study by Christiana, B *et al*. (15) shows that the CAMBRA method, in its current form, can lead to overtreatment. Among the limitations of this study are the lack of knowledge about caries activity and the risk of caries in the patient. The study is limited to the isolated and specific evaluation of the clinical case, leaving the diagnosis and treatment to the observer based only on clinical and radiographic observation.

Caries lesions are the main etiology in the indications of invasive dental treatment, the most frequent active intervention being tooth filling (16,17). The international caries consensus of 2016 led to clinical recommendations for the management of cavitated caries lesions and the removal of carious tissue, including restoration, based on the texture of demineralized dentin. Dentists must treat dental caries disease and control the activity of existing cavity lesions to preserve hard tissue and retain teeth over the long term. Entering the restoration cycle should be avoided as far as possible (18). The scientific evidence and therefore the recommendations given support less invasive management of caries lesions, delaying entry and slowing the restoration cycle by preserving dental tissue and retaining teeth in the long term (6). Based on these recommendations in the clinical case presented in this research, in the lesions of pits and fissures (Fig. 1) no invasive restorative treatment should be performed since no tooth has a manifest cavitated lesion on the occlusal faces; however, 22.0% of the respondents indicated restorative treatment of 46 and 47, only 17.0% indicated remineralization treatment, limiting 40.0% to clinical control of the lesions without any treatment. The professional application of different types of fluoride preparations has been shown to be effective in remineralizing caries in enamel (5% sodium fluoride) and dentine (38% silver diamine fluoride) (19,20). However, this was indicated by a minority of respondents. It is important to know that conservative remineralizing treatments based or not on fluoride and enamel regeneration offer a promising future for the control of early lesions (21). Evidence of the effectiveness of sealing the occlusal surfaces to reduce the incidence of caries has also been shown in the scientific literature (22,23), although invasive or non-invasive pit and fissure sealing was indicated by only 1.5% and 5.5% respectively. The results of our research show that the new knowledge and concepts on the management of caries lesions have not yet been incorporated by the surveyed dentists in their daily clinical practice, results that are consistent with other studies focused on different aspects of caries management, such as the research by Crespo-Gallardo *et al*. (24,25) on the diagnosis and treatment of deep caries, concluding that the recommendations based on scientific evidence in caries management are not yet part of dentists’ common clinical behavior.

The results of the study in the analysis of the radiographic image (Fig. 2) located caries lesions in 44 distal (1. 6%), 45 mesial (3.0%), 45 distal (96.0%), 46 mesial (100.0%), 46 distal (96.0%), 47 mesial (86.0%) and 47 distal (9.0%). There is unanimity of criteria in the identification of caries in 46 mesial and practically total agreement in 45, 46 distal and 47 mesial. Research on the detection of demineralization lesions in proximal areas by radiographic study shows low sensitivity (false negatives) of the professionals in their detection, making training of the clinicians necessary to improve the diagnosis (26,27). Likewise, it is a test with relative specificity (false positives) (28). The use of artificial intelligence systems with networks of neurons has offered a more precise diagnosis than that of clinical experts (29), as cervical areas that present less contrast are confused with demineralization lesions compatible with caries (30), as is the case in this research in lesions diagnosed at 44 distal, 45 mesial and 47 distal. To avoid misdiagnosis, alternative methods may be appropriate. The recently published meta-analysis (31) evaluating the accuracy of near-infrared light transillumination (NILT) compared to bitewing radiography (BW) for the detection of interproximal dental caries in the permanent dentition concludes that NILT has reasonably comparable accuracy to BW with 92.3% accuracy. Sensitivity was 0.97, and specificity was 0.91 with high certainty of the evidence. Other results when comparing the two methods do not demonstrate significant differences between the two methods (32), so clinicians’ knowledge of dental anatomy and its radiographic manifestation is important to differentiate caries-compatible demineralization lesions with the lower physiological contrast in the cervical area.

Invasive intervention by means of Black’s class II cavity preparation and obturation is the traditional treatment of caries lesions on the proximal faces. Current recommendations, however, such as the guide published by the Japanese Society of Conservative Dentistry (10), indicate restorative intervention only when radiographic examination reveals that the lesion affects more than one third of the dentin. In lesions limited to enamel and the external third of the dentin we can opt for non-invasive remineralization treatments with the application of fluoride and improved cleaning techniques in proximal areas (silk thread), or microinvasive treatments consisting of sealing or resin infiltrations that have shown better results than professional non-invasive treatment in the control of caries in these areas (33,34). In this research only 3.0% of the dentists indicated this microinvasive treatment, which shows the surveyed dentists are not up to date in the new management of caries lesions. Despite the fact that 92.8% of those surveyed perform conservative dentistry in their daily practice and 82.5% recognize the application of minimal intervention concepts, 99.0% indicated fillings with Black’s class II cavities, 3.0% indicate remineralizing treatments and 4.0% indicate radiographic control of the proximal areas. The concept of minimal intervention implies the philosophical concept of disease prevention and the preservation of dental structure (35-37); only with our change of attitude and patient education will we avoid over-treatment (38-41).

In health sciences, the change in clinical behavior of professionals in scientifically proven situations is slow, and dentistry is no exception (1,42). Caries is a disease for which the clinician is highly resistant to change. The results of this research show an attitude based on very interventionist criteria and with very little indication of non-invasive or micro-invasive treatments. However, we must avoid the restoration cycle and limit invasive treatments to only when there is no other treatment option, with the aim of lengthening the life of the tooth (43).

## Conclusions

1. Significant differences in the diagnosis of pit and fissure caries in 46 were found regarding dentists’ qualifications, between those with a “Degree” and those with a “Doctorate”.

2. No significant differences were found in the diagnosis of caries lesions in pits and fissures in relation to the years of professional practice, daily practice of conservative dentistry or the application of concepts of minimal intervention.

3. There were significant differences between the “Not applicable” group and the “Yes applicable” group in the treatment in the Filling of pits and fissures in 46 and 47 in terms of whether the dentists applied the concepts of minimal intervention in their treatments.

4. A significant difference was found between the Yes” and “No” groups in the treatment of pits and fissures with clinical control in terms of the dentist’s daily practice of conservative dentistry.

5. No significant differences were found in the treatment of caries lesions in pits and fissures in relation to the dentists’ qualifications or the years of professional practice.

6. No significant differences were found in the diagnosis or in the indications for treatment of proximal surfaces in relation to the dentist’s qualifications, years of professional practice, daily practice of conservative dentistry or the application of minimal intervention concepts.
